# Interaction Tolerance Detection Test for Understanding the Killing Efficacy of Directional Antibiotic Combinations

**DOI:** 10.1128/mbio.00004-22

**Published:** 2022-02-15

**Authors:** Jia-Feng Liu, Orit Gefen, Zi-Yin Zhang, Min-Min Liu, Maskit Bar-Meir, Nathalie Q. Balaban

**Affiliations:** a Racah Institute of Physics, Edmond J. Safra Campus, The Hebrew University of Jerusalem, Jerusalem, Israel; b Center for Infectious Disease Research, School of Medicine, Tsinghua University, Beijing, China; c Paediatrics and Infectious Diseases Division, Shaare Zedek Medical Center, Jerusalem, Israel; d Faculty of Medicine, The Hebrew University, Jerusalem, Israel; California Institute of Technology

**Keywords:** antibiotic persistence, antimicrobial activity, antimicrobial agents, antimicrobial combinations, synergy, tolerance

## Abstract

Combination treatments are commonly prescribed for enhancing drug efficacy, as well as for preventing the evolution of resistance. The interaction between drugs is typically evaluated near the MIC, using growth rate as a measure of treatment efficacy. However, for infections in which the killing activity of the treatment is important, measurements far above the MIC are needed. In this regime, the killing rate often becomes weakly concentration dependent, and a different metric is needed to characterize drug interactions. We evaluate the interaction metric on killing using an easy visual assay, the interaction tolerance detection test (iTDtest), that estimates the survival of bacteria under antibiotic combinations. We identify antibiotic combinations that enable the eradication of tolerant bacteria. Furthermore, the visualization of the antibiotic interactions reveals directional drug interactions and enables predicting high-order combination outcomes, therefore facilitating the determination of optimal treatments.

## INTRODUCTION

Drug cocktails, i.e., combinations of different drugs, are widely used for antibiotic treatments, as well as for anticancer therapy (1–4). Combinations were shown to improve curative effect, decrease toxicity and prevent the evolution of drug insusceptibility, such as resistance and tolerance (5–8). However, the determination of the way two drugs interact can be difficult and ambiguous (9–16).

A common method for detecting the effect of antibiotics combinations is the fractional inhibition concentration (FIC) index, which measures the effect of each treatment, as well as of the combination, on the growth rate of bacteria (17). To enable growth, the concentrations are required to be below the MIC. However, in current clinical practice, high concentrations, far above the MIC and often bactericidal, are typically used in order to cure persistent infections in immunocompromised patients (18) and to prevent the evolution of resistance (19), while interactions between drugs near MIC may differ when tested at bactericidal concentrations. For example, the combination for daptomycin (DAP) and rifampicin (RIF) is one of the possible treatments for bloodstream infections caused by methicillin-resistant Staphylococcus aureus (MRSA) (20). This combination is typically reported as neutral or synergistic by FIC test (21, 22). However, we recently identified that this combination is antagonistic under clinically relevant bactericidal concentration (8), in concordance with other reports (22). Similar discrepancies between the classifications of interactions below and above MIC were broadly reported (23–28), showing that knowledge on the interaction between drugs near the MIC may not always be predictive for the way they interact far above the MIC, which may be a relevant concentration for patients. Another difficulty stems from the fact that, even when a drug combination efficacy is evaluated on a strain, variants of that strain may change the way drugs interact (29). Such changes, involving tolerant and antibiotic persistent mutants, have recently been observed to occur during clonal evolution within patients (8, 30, 31).

Whereas the growth rate can be easily measured in parallel for many conditions, killing curves, which are needed for evaluating the killing efficacy, are significantly more difficult to perform. Thus, in order to minimize the inappropriate use of antibiotic combinations and to identify the cases where they are needed compared to monotherapy (32, 33), a method for an easy evaluation of the killing effect of combination treatments directly on isolates in the clinical environment is required (34, 35).

Here, we report the use of a modification of a tolerance detection test (34), the interaction tolerance detection test (iTDtest), for a semiquantitative evaluation of the killing efficacy of antibiotics combinations. Based on this test, we were able to predict the effect of high-order antibiotic combinations on killing and find combination treatments which can effectively kill the sensitive ancestral strain, as well as a tolerant mutant derivative.

## RESULTS

### Evaluation of the antibiotic combination killing efficacy with the iTDtest.

The Kirby-Bauer antibiotic disk diffusion assay is a well-established and robust method for estimating the MIC of bacteria by measuring the diameter of the inhibition zone (36). However, this assay does not distinguish between drugs that merely inhibit the growth or that kill bacteria. Among recent techniques for evaluating killing (37), the TDtest is a simple and easy modification of the disk diffusion assay that allows an evaluation of the killing efficiency of antibiotics (34). Briefly, the TDtest consists of two steps. In step I, bacteria are exposed to a disk with an amount of antibiotic low enough so that the concentration of antibiotic within the inhibition zone drops below MIC by diffusion after about a day. Once the concentration is below the MIC, surviving bacteria are able to form colonies. However, by this time, nutrients in the inhibition zone are depleted by the bacteria growing around it. Therefore, the TDtest step II consists of adding nutrients (glucose and amino acids) which allow surviving bacteria to grow to a visible colony (see Fig. S1A in the supplemental material). Thus, the TDtest reveals the hidden survival information present in the inhibition zone. The numbers of colonies that appear after step II (see Fig. S1A and B) provide a semiquantitative evaluation of survival and agree well with killing assays (see Fig. S1C). Examples of the TDtest performed with single drugs and its comparison with killing assays can be found in Fig. S1 and S^2^ and in previously published studies (8, 34, 38).

10.1128/mbio.00004-22.1 FIG S1 Semiquantitative evaluation of killing efficacy of antibiotics with the TDtest. (A) TDtest of AMP (100 μg) with 5 × 10^6^ bacteria plated. Step I, before addition of nutrients; step II, after addition of nutrients (34). Results for both E. coli wild-type (wt, KLY) and tolerant (*tol*, KLY-*metG^T^*) strains are shown. (B) Same as panel A but with 5 × 10^5^ bacteria plated. The number of surviving colonies increased in proportion to the number of bacteria plated, showing that the TDtest is a semiquantitative measurement of survival and in agreement with the corresponding killing assays shown in panel C. (C) Killing assay AMP, 100 μg/mL, 24 h). The data are presented as means ± the standard deviations from at least three biological replicates. Download FIG S1, PDF file, 0.2 MB. Copyright © 2022 Liu et al. 2022 Liu et al. https://creativecommons.org/licenses/by/4.0/ This content is distributed under the terms of the Creative Commons Attribution 4.0 International license.

**FIG 1:**
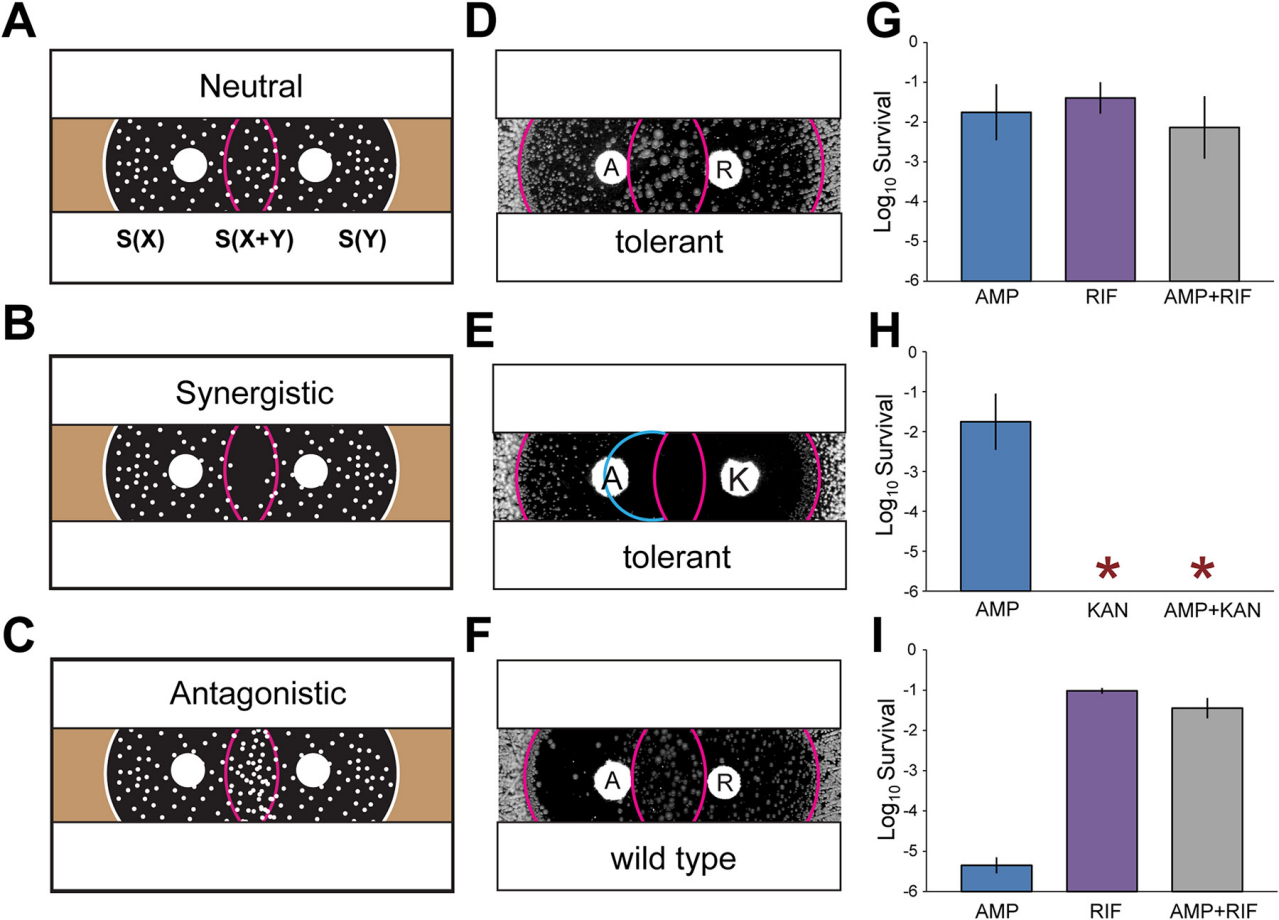
The iTDtest reveals drug interactions on killing and agrees with killing assays. (A to C) Schematic view of possible outcome of the iTDtest for detecting neutral, synergistic, or antagonistic interactions. (D to F) Examples of measurements of the three types of interactions with the iTDtest. (G to I) Corresponding killing assays in batch cultures (24 h) done for the same drug interactions shown in panels D to F. (D and G) AMP (A) and RIF (R) for tolerant strain. (E and H) AMP (A) and KAN (K) for tolerant strain. Note that the inhibition radius extends beyond the KAN inhibition, revealing an additional synergy (cyan curve in panel E). (F and I) AMP and RIF for the wild type. The iTDtest was performed in two steps (see Fig. S4), first, by exposing bacteria to the antibiotic disks (AMP, 100 μg; KAN, 15 μg; RIF, 200 μg), which resulted in a zone of inhibition, and then by the addition of a nutrient disk (5 μL of 40% glucose and 5 μL of 20% Casamino Acids) after 24 h. Killing can be evaluated by the number of surviving colonies inside the inhibition zone. Survival data are presented as means ± the standard deviations from at least three biological replicates. Synergy and suppression are defined according to the scheme shown in Fig. 3A to D. Wild type, E. coli KLY; tolerant, E. coli KLY-*metG^T^*.

In order to measure the killing efficacy of the combination of two drugs and compare it to each drug separately within the same assay, we performed the drug interaction TDtest (iTDtest). This text consists of using two disks impregnated with different antibiotics and placed at an appropriate distance so that mainly the region between the disks is exposed to the drug combination, whereas the external regions are effectively exposed to a single drug each. Three possible outcomes of the test, depending on the way the two drugs interact, are shown schematically in Fig. 1A to C. The interaction can be deduced from the comparison in the inhibition zone in the region between the disks and the external regions on the sides of each disk. If the survival, i.e., the number of microcolonies, in the intersect region is substantially decreased compared to that of a single drug, then this suggests a synergistic interaction, whereas an increased number of colonies in the intersect region implies antagonism. Note that the interaction that we measure is on the killing activity, in contrast to more conventional multiple disks tests that detect growth inhibition such as the d-test (39).

10.1128/mbio.00004-22.2 FIG S2 TDtest of KAN (A, 10 μg), RIF (C, 100 μg), and killing assay after 24 h (B, KAN, 30 μg/mL; D, RIF, 200 μg/mL). (A and C) Step I, before addition of nutrients; step II, after addition of nutrients. The tolerant strain had a similar high survival as wt under RIF and a similar low survival as wt under KAN. There was a quantitative correspondence of the survival determined by killing assay in liquid medium and the TDtest. Results for both E. coli wild-type (wt, KLY) and tolerant (*tol*, KLY-*metG^T^*) strains are shown. The data are presented as means ± the standard deviations from at least three biological replicates. *, Below detection limit. Download FIG S2, PDF file, 0.2 MB. Copyright © 2022 Liu et al. 2022 Liu et al. https://creativecommons.org/licenses/by/4.0/ This content is distributed under the terms of the Creative Commons Attribution 4.0 International license.

10.1128/mbio.00004-22.3 FIG S3 MIC of AMP, KAN, and RIF by microdilution. The MICs of E. coli wild-type (KLY) and tolerant (KLY-*metG^T^*) strains were almost identical. Brown wells, growth; white wells, no growth. Defined as follows: for KAN, net increase of OD_600_ < 0.2 after 24 h; for others, net increase of OD_600_ < 0.05 after 24 h). Numbers in the well = net increase of OD_600_. The mean results for three biological replicates are shown. Download FIG S3, PDF file, 0.1 MB. Copyright © 2022 Liu et al. 2022 Liu et al. https://creativecommons.org/licenses/by/4.0/ This content is distributed under the terms of the Creative Commons Attribution 4.0 International license.

10.1128/mbio.00004-22.4 FIG S4 iTDtest detects the effect on killing of various antibiotic combinations. (A, D, and G) AMP and KAN. The cyan curve marks the enlarged region of killing due to a small synergistic effect. (B, E, and H) AMP and RIF. (C, F, and I) KAN and RIF. For KAN and RIF, the disks were placed on the agar plate one day before plating the bacteria to achieve a larger radius of inhibition. Representative results for E. coli wild-type (KLY) and tolerant (KLY-*metG^T^*) strains are shown, and all experiments were repeated with biological triplicates. For killing assays, AMP (100 μg/mL, ∼20× MIC), KAN (30 μg/mL, ∼7.5× MIC), and RIF (100 μg/mL, ∼10× MIC). Survival was measured after 24 h. The data are presented as means ± the standard deviations from at least three biological replicates. *, Below detection limit. Download FIG S4, PDF file, 0.3 MB. Copyright © 2022 Liu et al. 2022 Liu et al. https://creativecommons.org/licenses/by/4.0/ This content is distributed under the terms of the Creative Commons Attribution 4.0 International license.

We performed iTDtests for ampicillin plus kanamycin (AMP+KAN), AMP+RIF, and KAN+RIF on wild-type (wt) and tolerant E. coli strains and compared the interaction identified with killing assays (see Fig. S4). Notably, interactions agreed well between the killing assay and the iTDtest. For example, AMP+RIF is strongly antagonistic in the iTDtest (Fig. 1F), as well as in the killing assay (Fig. 1I), where adding RIF decreases the killing effect of AMP on the wt strain (40).

The iTDtest showed that survival in the combination region is often largely identical to that in the one component drug region. For example, iTDtesting of tolerant strains for AMP+KAN and RIF+KAN showed no colony in the combination region, as well as in the region of KAN alone (Fig. 1E; see also Fig. S4C). This suggests that a killing concentration of KAN may be a potent antitolerant drug even when KAN is used alone (41).

**FIG 2:**
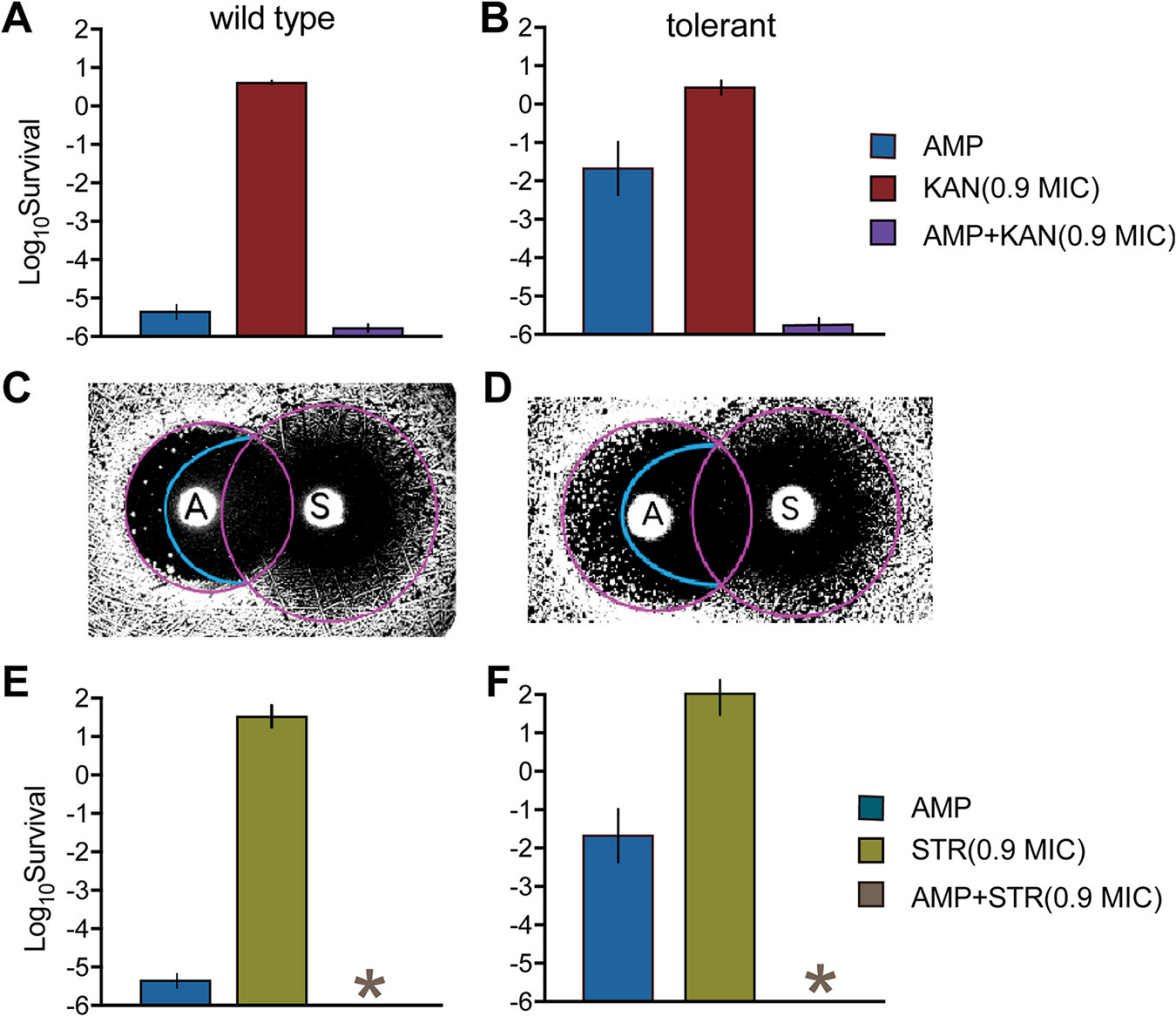
Killing assays confirm the strong synergy detected with the iTDtest. (A and B) Killing assay in liquid culture with AMP (100 μg/mL) + KAN (3.6 μg/mL). Survival was measured after 24 h. (C and D) iTDtest with AMP (100 μg) and STR (30 μg). The cyan lines mark the region of synergy beyond the initial radius of inhibition, suggesting synergy below MIC of STR. (E to F) Killing assays in liquid culture with AMP (100 μg/mL) + STR (7.2 μg/mL) confirm the synergy revealed by the iTDtests in panels C and D. Survival was measured after 24 h. Synergy and suppression are defined according to the scheme shown in Fig. 3A to D. The data are presented as means ± the standard deviations from at least three biological replicates. Wild type, E. coli KLY; tolerant, E. coli KLY-*metG^T^*; *, below detection limit.

Strikingly, careful examination of the effect of AMP+KAN on the tolerant strain reveals a synergistic effect, as shown by an increased efficiency of killing even beyond the inhibition zone of KAN (Fig. 1E, cyan line). This implies that KAN is able to reduce the AMP persistence level even beyond its killing ability, thus preventing the appearance of the microcolonies even beyond the KAN inhibition radius. We note that this synergistic effect cannot be seen in the killing assay with a high concentration of KAN because of its detection limit (Fig. 1H). To confirm this synergistic interaction, we performed killing assays of AMP combined with a sub-MIC of KAN. Remarkably, although bacteria could grow at 0.9× MIC of KAN, a combination killing assay of AMP+KAN (0.9× MIC) could substantially kill persisters (Fig. 2A and B). In order to test the generality of the result, we tried another aminoglycoside antibiotic, streptomycin (STR). The results obtained with an iTDtest and a killing assay with AMP+STR were similar to those obtained with AMP+KAN, showing that the iTDtest can detect the synergistic killing of AMP with a sub-MIC of STR, reducing further the survival of AMP persisters (Fig. 2C to F). These results show that the iTDtest can be used to directly visualize and detect synergistic and antagonistic effects of combination treatments on killing efficacy, above and below MIC.

### Interactions in a strongly cidal regime reveals the directionality of drug interactions.

So far, we have considered a drug interaction as synergistic when the killing achieved was higher than with any of the two drugs alone; similarly, antagonistic interactions are defined as a lower level of killing than that obtained with either drug alone. Here, we define more quantitatively the notion of drug interactions in the killing regime at a high drug concentration. To evaluate drug interactions, definitions for “effect independence” (9) and “concentration additivity” (10) were proposed by Bliss and Loewe, respectively. Loewe additivity suggests that for a neutral combination, the two drugs would be interchangeable and that the combination of drug A (at concentration *c*_A_) and drug B (at concentration *c*_B_) would have the same effect as the use of either drug A or B alone at concentration *c*_A_ +* c*_B_. On the other hand, Bliss’ null hypothesis assumes that two drugs function independently and that the effect of the combination is *E*_AB_^ ^= *E*_A_ × *E*_B_, where *E* is the effect on survival (15). Loewe’s assumption become irrelevant in the regime of high antibiotic concentrations, where the killing effect becomes quasi-independent of the drug concentration. This explains why the FIC test, which is based on the concentration additivity hypothesis, may fail at predicting interactions at higher drug concentrations and in combinations with more than two drugs (15). When analyzing multiple combinations using the Bliss’ model, few combinations could be defined as synergistic or even neutral with respect to their killing efficacy at high concentrations (8, 27), suggesting that this hypothesis of independency fails at high killing concentrations.

In order to understand the high concentration range, it is useful to consider the effect of the concentration on killing, which can be modeled with the modified Zhi function:

$$\varphi \left(c\right)=\frac{\text{ln}(0.01)}{\mathrm{MD}K_{99}}\cdot \frac{1-{\left(\frac{c}{\mathrm{MIC}}\right)}^{k}}{\frac{\text{ln}(0.01)}{\varphi_{\max}\times \mathrm{MD}K_{99}}-{\left(\frac{c}{\mathrm{MIC}}\right)}^{k}}$$

Here, *φ* is the growth rate when positive and kill rate when negative (42–44), *c* is the drug concentration, *MDK_99_* is the minimum duration for 99% killing, and *k* is the Hill coefficient. The effect of a drug becomes concentration insensitive when the concentration is much higher than the MIC (12). In this regime, the killing rate become independent of concentration at:

$$\varphi \left(c\right)∼\varphi_{\min}=\frac{\text{ln}(0.01)}{\mathrm{MD}K_{99}},$$

so that the survival after an exposure of duration (*t*), S(c,t)=exp(φ(c).t), is also independent of concentration. This analysis suggests that in this regime, the interactions need to be defined differently. First, the combination of a drug with itself, would be φ(c+c)≈φ(c), so that for two drugs A and B (A more potent than B), |φ(A+B)|≈|φ(A)| would be expected for an indifferent interaction (45).

**FIG 3:**
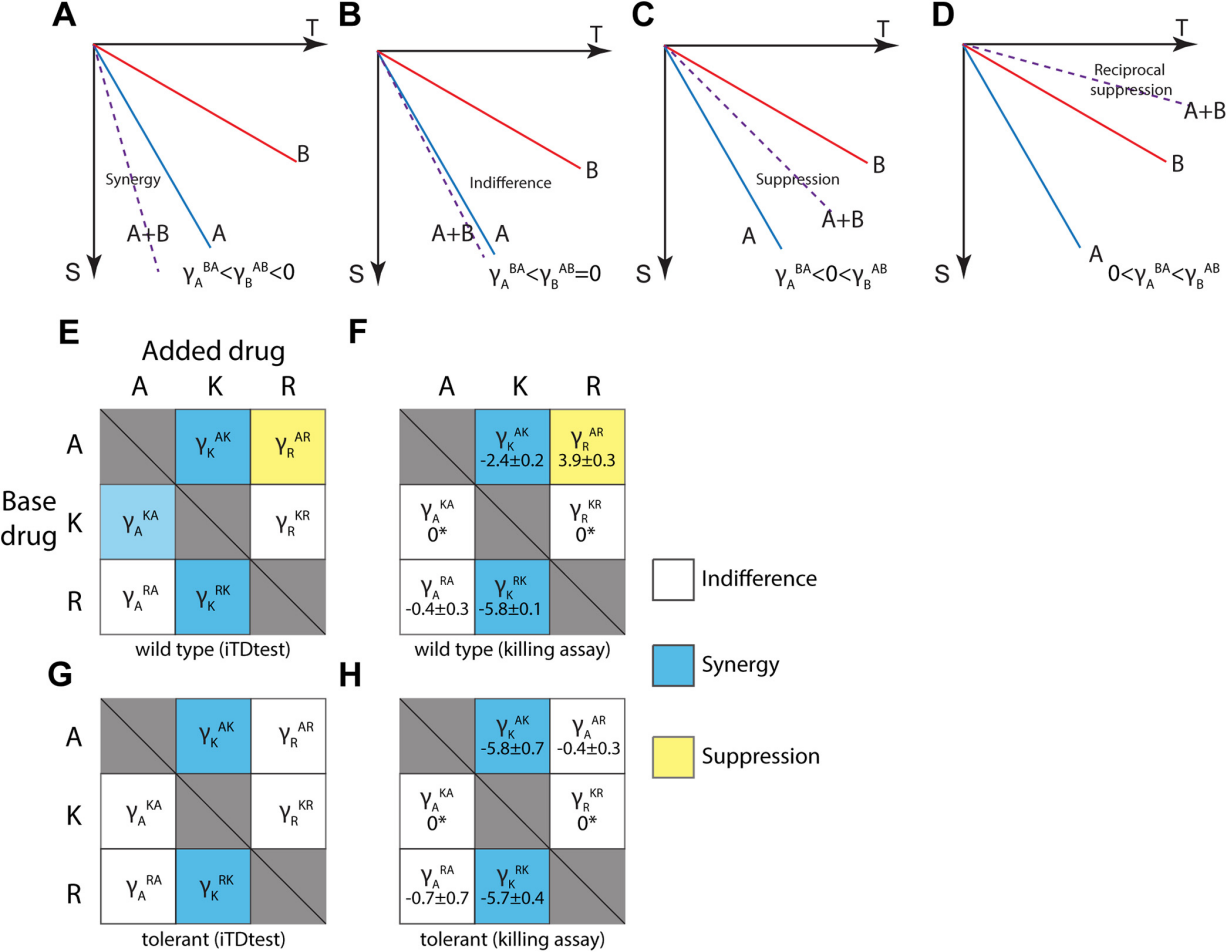
Matrices of interaction factors reveal direction of combination. (A to D) Schematic view for the different drug interaction classifications in the killing regime. S, log of survival; T, time. (E to H) Matrices of directional interaction factors $\gamma_{j}^{\mathrm{ij}}$. Each row is for a given drug (A, AMP; K, KAN; and R, RIF), and the matrix value determines the effect of adding a second drug according to column. Synergy means that adding the second drug enhances the killing, whereas suppression means that adding the second drug reduces the killing. (E and G) iTDtest; (F and H) killing assay. The numbers in the matrices represent the interaction factor ± the standard deviations calculated from survival. A γ value between −1 to 1 was defined as indifference. Asterisk, survival below detection limit. Wild type, E. coli KLY; tolerant, E. coli KLY-*metG^T^*.

To evaluate the effect shown by iTDtests or killing assays, and recognize the direction of combinations, we defined the combination factor $\gamma_{j}^{\mathrm{ij}}={\tilde{S}}_{\mathrm{ij}}-{\tilde{S}}_{i}$, where ${\tilde{S}}_{i}$, and ${\tilde{S}}_{\mathrm{ij}}$ are the absolute value of the log survival under drug *i*, and the combination of *i* and *j*, respectively. Intuitively, $\gamma_{j}^{\mathrm{ij}}$ represents the effect of drug *j* in the combination *ij*, once the effect of drug *j* is subtracted, and enables to classify the effect of drug combination according to the scheme of Fig. 3A to D. Note that usually $\gamma_{j}^{\mathrm{ij}}\neq \gamma_{i}^{\mathrm{ji}}$. For example, the effect of AMP on KAN is characterized by $\gamma_{A}^{\mathrm{KA}}$ = 0, which means that KAN killing is indifferent to the presence of AMP (within our detection limit) (Fig. 3F; see also Fig. S4). In contrast, the killing by AMP becomes more potent in the presence of KAN, as reflected by $\gamma_{K}^{\mathrm{AK}}$= –2.4 (Fig. 3F; see also Fig. S4). Applying the same classification scheme for the combination of AMP and RIF, with $\gamma_{R}^{\mathrm{AR}}$ = 3.9 and $\gamma_{R}^{\mathrm{AR}}$ = –0.4, shows that RIF suppresses AMP killing. The small value of $\gamma_{R}^{\mathrm{AR}}$ suggests that the survival under the combination is largely determined by RIF. We regroup the data obtained using the iTDtest and killing assay for the wt and tolerant strains of E. coli in interaction matrices of combination factors (Fig. 3E to H). These matrices demonstrate a few interesting characteristics of drug combinations in the strongly cidal regime. First, we see very good agreement between the classifications obtained using the iTDtest results and the killing assays (Fig. 1 and 2; see also Fig. S4). Second, some differences were observed between wt and tolerant strains showing that combinations that are effective for wt strains may fail on tolerant strains. Third, in contrast to typical interaction measures such as the FIC, these matrices are not symmetric and reveal the directional interactions. These matrices then address the benefit of adding a second drug, given than a single drug is already administered, which can be relevant when switching from mono to combination therapy, as is often the case. For example, given that a patient is treated with AMP as “base drug” (first row in the matrix of Fig. 3E), adding KAN is beneficial ($\gamma_{K}^{\mathrm{AK}}<-1$) , whereas adding RIF is detrimental $(\gamma_{R}^{\mathrm{AR}}>1$)  to the killing efficacy of the treatment. In contrast, if the patient is under KAN treatment as “base drug” (second row in the matrix of Fig. 3E), adding either AMP or RIF will hardly affect the killing efficacy of the treatment. Specifically, $\gamma_{A}^{\mathrm{KA}}\approx \gamma_{R}^{\mathrm{KR}}\approx 0$ is in agreement with the neutral hypothesis, where the most potent drug (KAN) determines the effect of the combination.

### Prediction of higher-order combinations.

**FIG 4:**
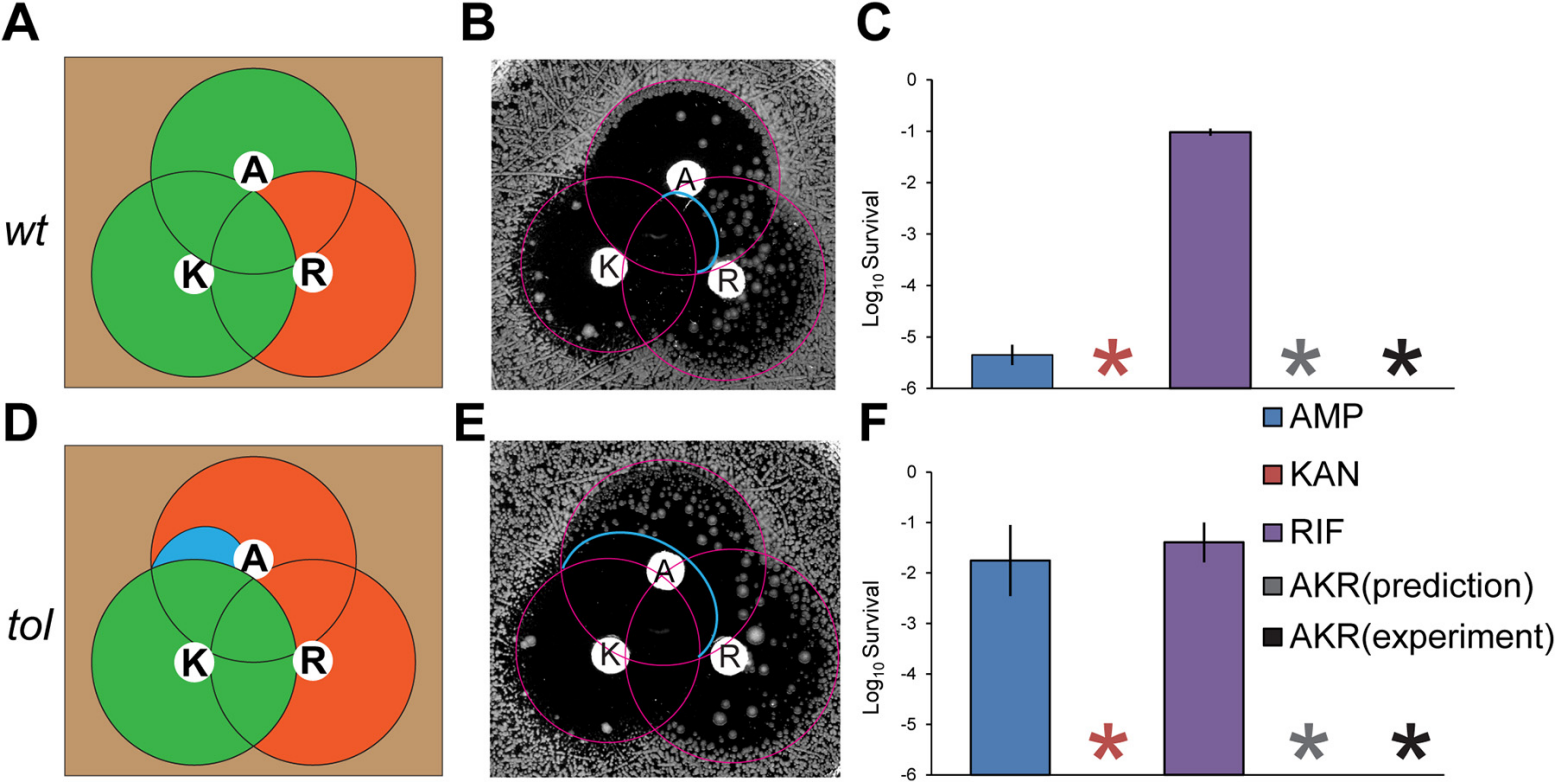
TDtest and killing assay with triple antibiotic combination. Based on double iTDtest results (RIF dominates AMP, and KAN dominates AMP and RIF), we can predict the triple interaction of AMP, KAN, and RIF that the effect of KAN would dominate. (A and D) Prediction. Green, predicted clean regions; orange, predicted regions with surviving colonies; cyan, predicted clean regions because of small synergistic effect by AMP. (B and E) Experimental validation of the predictions. (AMP, 100 μg; KAN, 15 μg; RIF, 200 μg). (C and F) Killing assay with a triple antibiotic combination after 24 h. AMP (100 μg/mL, ∼20× MIC), KAN (30 μg/mL, ∼5× MIC), RIF (100 μg/mL, ∼10× MIC). Gray, prediction; black, experiment. wt, E. coli KLY; *tol*, E. coli KLY-*metG^T^*; *, below detection limit.

The interaction matrices for two-drug combinations obtained from the iTDtest and shown in Fig. 3 enable to predict the result of combining the three drugs. Specifically, because $\gamma_{A}^{\mathrm{KA}}\approx \gamma_{R}^{\mathrm{KR}}\approx 0$, i.e., both RIF and AMP do not affect KAN killing, the killing under the three drugs should be dominated by KAN. The prediction would be φ(A+K+R)≈φ(K), or $\gamma_{\mathrm{AR}}^{\mathrm{KAR}}\approx 0$. Indeed, the mode of combination revealed from the triple iTDtest (Fig. 4B and E) or killing assay under the three drugs (Fig. 4C and F) was consistent with this prediction.

In addition, the iTDtest can reveal subtler interactions. For example, we could predict that the small synergistic effect of AMP on KAN revealed by the iTDtest for the tolerant strain (Fig. 1E, cyan curve) would also appear on the triple iTDtest as a depletion in the surviving colonies beyond the inhibition radius of KAN (cyan curve, Fig. 4E). Moreover, beyond the inhibitory ring of KAN, there was a clean area without colonies growing, suggesting that the small synergistic effect of KAN to AMP was kept under the triple combination, even when the concentration of KAN was below MIC. Thus, the iTDtest reveals that the triple combination AMP+KAN+RIF or double combination AMP+KAN could be potent against tolerant bacteria. We conclude that our directional two-drug interaction classification can help predict higher-order interactions. We note that predictions could be made here because of the simplicity of the double combinations.

Due to the limitation in the number of disks that fit in the interaction area, an iTDtest with more than three antibiotic disks would be too difficult to analyze. In order to allow testing of more than three drugs and make this method scalable, we combined two drugs in one disk, testing the interaction of the two-drug disk with a third drug on a separate disk. We performed the iTDtest with this strategy and found that the results fit well with our previous results (Fig. 4F; see also Fig. S5). We conclude that the iTDtest performed on two-drug combinations enable to predict the three-drug combination and reveals the strong potency of the AMP+RIF+KAN combination on tolerant strains (see Fig. S5).

10.1128/mbio.00004-22.5 FIG S5 iTDtests performed by grouping two antibiotics in one disk reveal the same pattern as the three-disks iTDtest. (Upper panel) TDtest for combinations of two or three antibiotics. (Lower panel) Instead of using three separate disks, as in Fig. 4, two antibiotics can be combined into a single disk, and the iTDtest was performed using an additional disk. Thus, any number of antibiotics can be combined to perform the iTDtest. Results for E. coli wild-type (wt, KLY) or tolerant (*tol*, KLY-*metG^T^*) strains are shown. Download FIG S5, PDF file, 0.2 MB. Copyright © 2022 Liu et al. 2022 Liu et al. https://creativecommons.org/licenses/by/4.0/ This content is distributed under the terms of the Creative Commons Attribution 4.0 International license.

## DISCUSSION

By measuring the killing efficacy of antibiotics combinations with the iTDtest, we were able to easily identify antibiotic combinations with synergistic effects on killing and, in particular, against tolerant bacteria. The results show that the iTDtest can reproduce the interactions measured by killing assays in liquid culture. Killing assays are better at quantifying survival but are difficult to perform on many different antibiotic concentrations and exposure durations. Thus, the iTDtest may be a useful tool for selecting the optimized drug regimen for hard-to-treat infections.

Since the evolution of tolerance has been shown to be a stepping stone for the evolution of resistance (8, 35), combatting the evolution of tolerance is of high priority, especially for persistent infections in immunocompromised patients (46). The low cost and easy detection of beneficial drug interactions at the clinically relevant concentration can be done for each strain isolated from patients along these hard-to-treat infections. Since such infections are often treated by adding sequentially drugs, our framework allows us to directly visualize and assess the effect of adding a drug on the killing efficacy compared to one drug alone, thus guiding toward the most effective combination treatment tailored to each specific infecting strain. Future improvement of the method could be done by automated detection and quantification of the regrowing colonies inside the inhibition zone.

The easy visualization of two-drug interactions with the TDtest also enabled to predict three-drug interactions for the few drugs tested. More drugs must be analyzed to assess the generality of this prediction and extend the framework to subtler interactions. Our results suggest new avenues for designing drug combinations, a goal with basic research and practical interests (47, 48). For antibiotics, for which the discovery of new compounds is becoming scarce (49), the many possibilities that multiple antibiotic combinations offer could be an invaluable source of effective treatments when tailored to the infecting strains.

## MATERIALS AND METHODS

### Growth conditions.

E. coli and S. aureus were inoculated into LB medium (BD) or cation-adjusted Mueller-Hinton broth (Sigma, Israel), respectively. For DAP-related experiments, 0.5 mM CaCl_2_ was supplemented to the medium (50). The bacteria were grown to stationary phase at 37°C with shaking overnight and stored in aliquots with 15% glycerol at –80°C.

### Strains and plasmids.

Clinical MRSA isolates P1D1C1 and P1D7C1 are ancestral and derived tolerant strains isolated from the same patient with persistent bloodstream infection, as described previously (8). E. coli strains KLY and KLY-*metG^tol^* are the ancestral and derived tolerant strain isolated from an *in vitro* experimental evolution experiment under cyclic ampicillin treatment, as described previously (35, 51).

### MIC measurement with microdilution.

Around 5 × 10^5^ bacteria from the stock aliquots were grown in liquid medium at 37°C for 24 h with different concentrations of antibiotics. The MIC value was read as the lowest concentration without detectable growth (for KAN, net increase of OD_600_ < 0.2 after 24 h; for AMP and RIF, net increase of OD_600_ < 0.05 after 24 h).

### MIC measurement with Etest.

Approximately 10^7^ bacteria from the stock aliquots were plated on medium agar, and one Etest strip (bioMérieux) was placed. The MIC value was read after incubation at 37°C for 24 h.

### Antibiotics killing assays.

Aliquots of bacteria were diluted 1:100 in medium with antibiotics or the combinations, incubated at 37°C with shaking. At certain time points, a fraction of the culture was centrifuged at 5,000 × *g* for 5 min and washed with 0.9% NaCl twice to remove the antibiotics. After plating on agar medium and incubating for 24 to 48 h, the CFU were counted. At least three biological independent experiments were done in all assays. For the detection limit, 100 μL of the culture was plated, containing 10^6^ CFU. This corresponds to a detection limit for survival of 10^−6^.

### TDtest.

The TDtest was performed in two steps as follows. Step I is Kirby-Bauer disk diffusion. Filter paper (Whatman, #1) was sterilized by autoclave and cut in disks 6 mm in diameter. Bacteria from frozen aliquots are diluted in saline solution and plated on solid agar medium, and then paper disks impregnated with antibiotic are placed. For KAN and RIF, antibiotic disks are placed on the agar medium at 4°C for 24 h prior to plating the bacteria to enable the prediffusion of the antibiotic and reach bigger radius of inhibition. The disks are then removed, and approximately 5** × **10^5^ bacteria are plated and incubated at 37°C.

In step II, antibiotic disks, after 20 to 24 h, are replaced with nutrition disks containing glucose (2 mg) and Casamino Acids (2 mg). The plates are incubated further at 37°C for growth of the tolerant colonies, and the nutrients are replenished every day by adding solution containing glucose (2 mg) and Casamino Acids (2 mg) to the disk. The results of step II are assessed after an additional 24 h, unless specified otherwise.

### Growth rate measurement.

Bacteria were grown in a 96-well plate with the monochromator Infinite plate reader (Tecan, Switzerland) at 37°C with shaking, with continuous monitoring of the OD_600_. The growth rate was calculated by fitting the exponential part of the growth for three biological replicates for each strain.

### Data availability.

Data are available at http://bio-site.phys.huji.ac.il/Materials.

10.1128/mbio.00004-22.6 TABLE S1 Strains. Download Table S1, PDF file, 0.2 MB. Copyright © 2022 Liu et al. 2022 Liu et al. https://creativecommons.org/licenses/by/4.0/ This content is distributed under the terms of the Creative Commons Attribution 4.0 International license.
