# Supplementary material for: Interaction Tolerance Detection Test for Understanding the Killing Efficacy of Directional Antibiotic Combinations
Source: mBio. 2022 Feb 15;13(1):e00004-22. doi: 10.1128/mbio.00004-22 (PMC8844919; doi:10.1128/mbio.00004-22)
Supplement: FIG S1 [file mbio.00004-22-sf001.pdf]

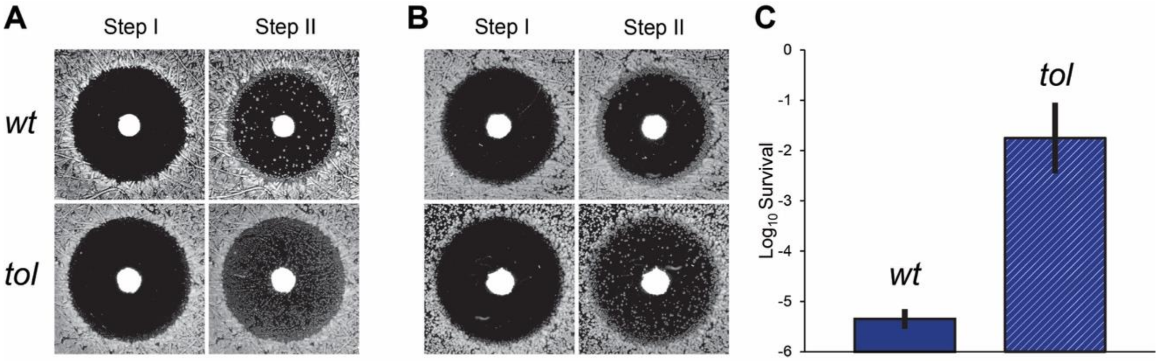

Fig. S1. Semi-quantitative evaluation of killing efficacy of antibiotics with the TDtest. (A) TDtest of AMP (100 µg) with 5×10^6 bacteria plated. Step I: before addition of nutrients; Step II: after addition of nutrients (34). Results for both *E. coli* wild type (*wt*, KLY) and tolerant (*tol*, KLY-*metG^T*) strains are shown. **(B) Same as (A) with , 5×10^5 bacteria plated.** The number of surviving colonies increased in proportion to the number of bacteria plated, showing that the TDtest is a semi-quantitative measurement of survival, and in agreement with the corresponding killing assays shown in (C). **(C) Killing assay AMP, 100 µg/ml, 24 hours).** Data are presented as the mean ± s.d. from at least three biological replicates.
