# Supplementary material for: Interaction Tolerance Detection Test for Understanding the Killing Efficacy of Directional Antibiotic Combinations
Source: mBio. 2022 Feb 15;13(1):e00004-22. doi: 10.1128/mbio.00004-22 (PMC8844919; doi:10.1128/mbio.00004-22)
Supplement: FIG S2 [file mbio.00004-22-sf002.pdf]

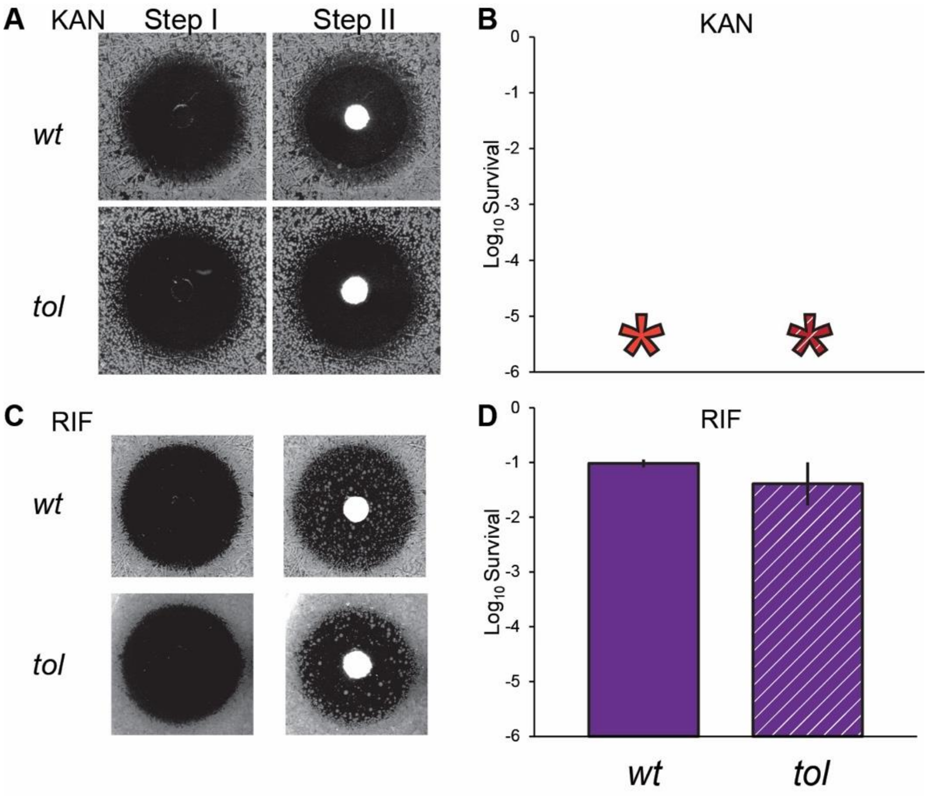

Fig. S2. TDtest of KAN (A, 10 µg), RIF (C, 100 µg) and killing assay after 24 hours (B, KAN, 30 µg/ml, D, RIF, 200 µg/ml). A, C, Step I: before addition of nutrients; Step II: after addition of nutrients. The tolerant strain had a similar high survival as *wt* under RIF, and a similar low survival as *wt* under KAN. There was a quantitative correspondence of the survival determined by killing assay in liquid medium and the TDtest. Results for both *E. coli* wild type (*wt*, KLY) and tolerant (*tol*, KLY-*metG*^{T}) strains are shown. Data are presented as the mean ± s.d. from at least three biological replicates. Asterisks: below detection limit.
