# Supplementary material for: Interaction Tolerance Detection Test for Understanding the Killing Efficacy of Directional Antibiotic Combinations
Source: mBio. 2022 Feb 15;13(1):e00004-22. doi: 10.1128/mbio.00004-22 (PMC8844919; doi:10.1128/mbio.00004-22)
Supplement: FIG S3 [file mbio.00004-22-sf003.pdf]

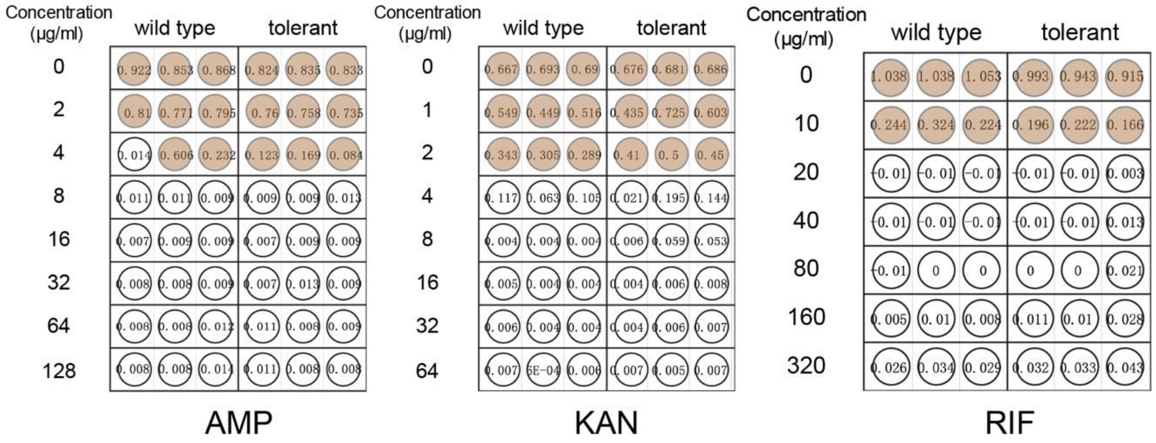

Fig. S3. MIC of AMP, KAN and RIF by microdilution. The MICs of *E. coli* wild type (KLY) and tolerant (KLY-$metG^T$) strains were almost identical. Brown wells: growth; white wells: no growth. Defined as: for KAN, net increase of OD_{600nm} < 0.2 after 24 hours; for others, net increase of OD_{600nm} < 0.05 after 24 hours). Numbers in the well: net increase of OD_{600nm}.Mean results of three biological replicates are shown.
