# Supplementary material for: Interaction Tolerance Detection Test for Understanding the Killing Efficacy of Directional Antibiotic Combinations
Source: mBio. 2022 Feb 15;13(1):e00004-22. doi: 10.1128/mbio.00004-22 (PMC8844919; doi:10.1128/mbio.00004-22)
Supplement: FIG S4 [file mbio.00004-22-sf004.pdf]

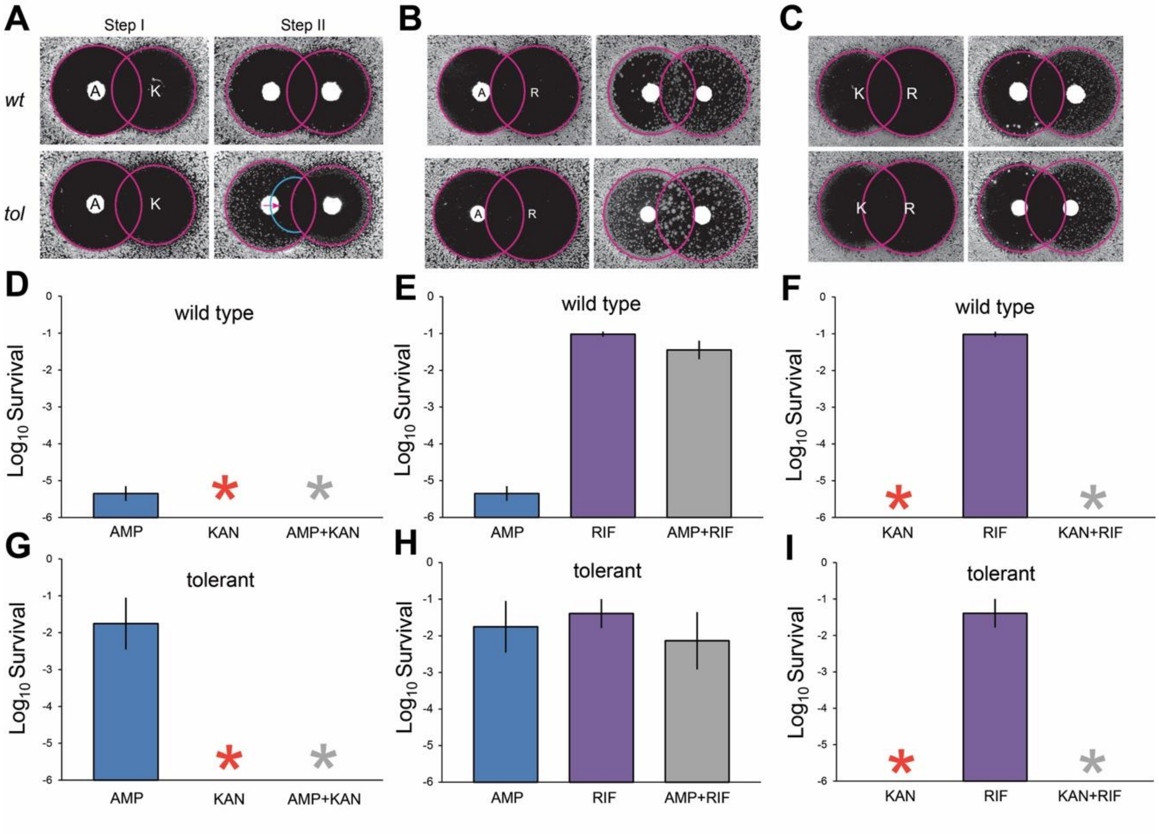

Fig. S4. iTDtest detects the effect on killing of various antibiotic combinations. (A, D, G) AMP and KAN. The cyan curve marks the enlarged region of killing due to a small synergistic effect. (B, E, H) AMP and RIF. (C, F, I) KAN and RIF. For KAN and RIF, the disks were placed on the agar plate one day before plating the bacteria to achieve a larger radius of inhibition. Representative results for *E. coli* wild type (KLY) and tolerant (KLY-*metG*^{T}) strains are shown, and all experiments were repeated with biological triplicates. For killing assays, AMP (100 µg/ml, ~20 x MIC), KAN (30 µg/ml, ~7.5 x MIC), RIF (100 µg/ml, ~10 x MIC). Survival was measured after 24 hours. Data are presented as the mean ± s.d. from at least three biological replicates. Asterisks: below detection limit.
