# Supplementary material for: Interaction Tolerance Detection Test for Understanding the Killing Efficacy of Directional Antibiotic Combinations
Source: mBio. 2022 Feb 15;13(1):e00004-22. doi: 10.1128/mbio.00004-22 (PMC8844919; doi:10.1128/mbio.00004-22)
Supplement: FIG S5 [file mbio.00004-22-sf005.pdf]

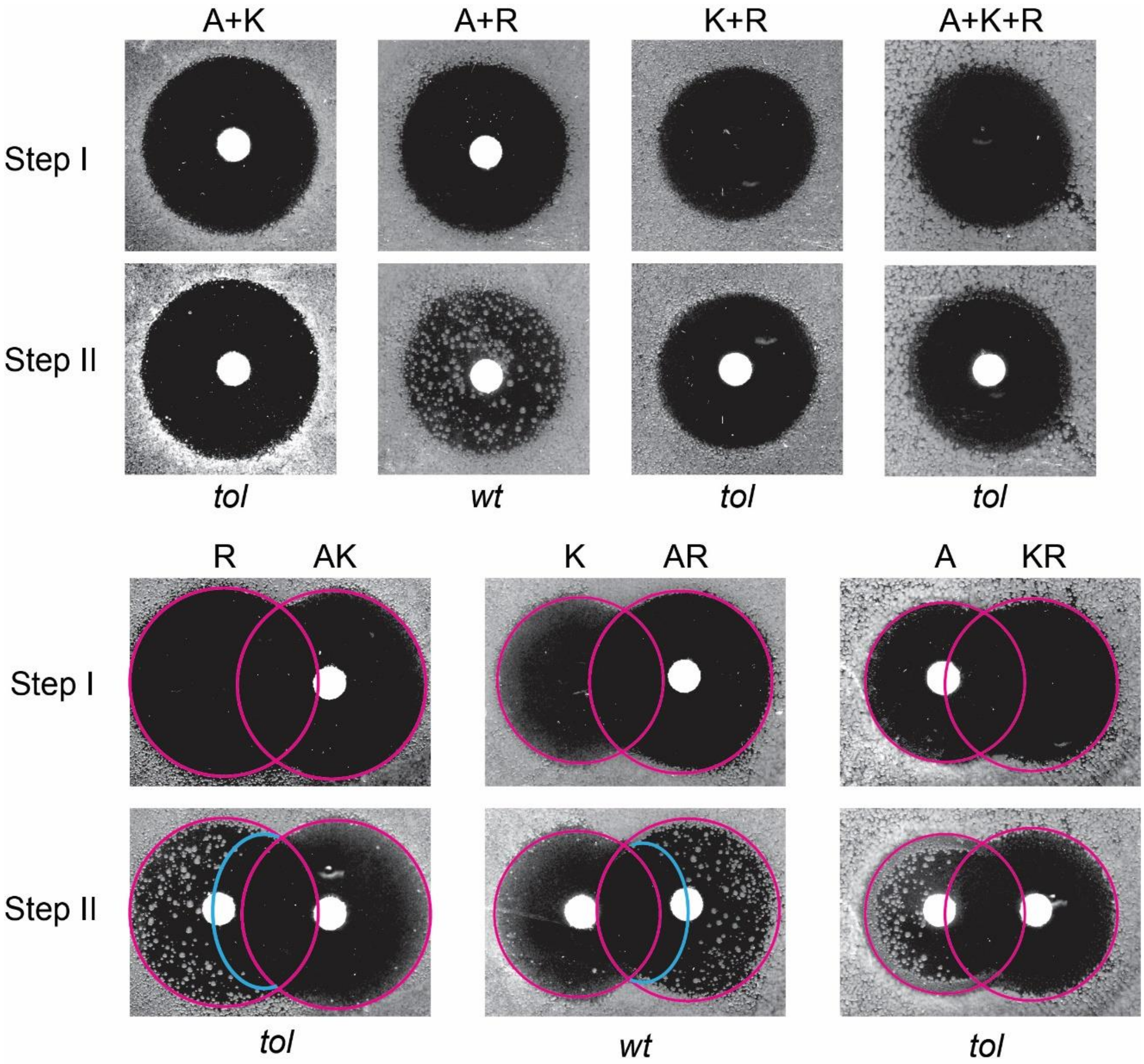

Fig. S5. iTDtests performed by grouping two antibiotics in one disk reveal the same pattern as the 3-disks iTDtest. Upper panel: TDtest for combinations of 2 or 3 antibiotics. Lower panel: instead of using three separate disks as in Fig. 4, two antibiotics can be combined into a single disk and the iTDtest performed using an additional disk. Thus, any number of antibiotics can be combined to perform the iTDtest. *E. coli* wild type (*wt*, KLY) or tolerant (*tol*, KLY-*metG*^{T}) strains.
