# Supplementary material for: Interaction Tolerance Detection Test for Understanding the Killing Efficacy of Directional Antibiotic Combinations
Source: mBio. 2022 Feb 15;13(1):e00004-22. doi: 10.1128/mbio.00004-22 (PMC8844919; doi:10.1128/mbio.00004-22)
Supplement: TABLE S1 [file mbio.00004-22-st001.pdf]

Table S1. Strains

Strain (strain name)	Construction	Genotype	Phenotype	Reference
**P1D1C1**	MRSA strain isolated from patient's blood		Ancestral	(1)
**P1D7C1**	MRSA strain isolated from patient's blood	P1D1C1 *rpoC rsgA*	VAN/DAP tolerant	(1)
**KLY**		KL16-YFP Cam	Ancestral	(2)
**KLY *metG*^{T}**	*In vitro* evolution	KLY *metG*-Δ569-572	AMP tolerant	(3, 4)

1. Liu J, Gefen O, Ronin I, Bar-Meir M, Balaban NQ. 2020. Effect of tolerance on the evolution of antibiotic resistance under drug combinations. Science 367:200-204.
2. Fridman O, Goldberg A, Ronin I, Shoresh N, Balaban NQ. 2014. Optimization of lag time underlies antibiotic tolerance in evolved bacterial populations. Nature 513:418-21.
3. Levin-Reisman I, Ronin I, Gefen O, Braniss I, Shoresh N, Balaban NQ. 2017. Antibiotic tolerance facilitates the evolution of resistance. Science 355:826-830.
4. Levin-Reisman I, Brauner A, Ronin I, Balaban NQ. 2019. Epistasis between antibiotic tolerance, persistence, and resistance mutations. Proceedings of the National Academy of Sciences of the United States of America 116:14734-14739.
